# Editorial: Crosstalk: skin cells and immune cells in inflammatory skin diseases

**DOI:** 10.3389/fimmu.2024.1472313

**Published:** 2024-08-23

**Authors:** Kazuhiko Yamamura, Hyun Je Kim, Jeong Eun Kim

**Affiliations:** ^1^ Department of Dermatology, Kyushu University, Fukuoka, Japan; ^2^ Department of Biomedical Sciences, Seoul National University Graduate School, Seoul, Republic of Korea; ^3^ Department of Dermatology, College of Medicine, Hanyang University, Seoul, Republic of Korea; ^4^ Hanyang Institute of Bioscience and Biotechnology, Hanyang University, Seoul, Republic of Korea

**Keywords:** skin, immune cells, crosstalk, inflammation, atopic dermatitis, psoriasis

The skin is the human body’s largest organ consisting of two layers: epidermis and dermis, and appendages: hair and sweat glands. The skin not only wraps the body but also protects it from external stimuli and infection, perceives sensations such as pain and itch, and coordinates with various circulating immune cells for immune response/regulation. Recent studies have shown that inflammatory skin diseases, including psoriasis and atopic dermatitis, harbor systemic inflammation/immune abnormalities such as strong Th activation and expansion of specific immune cell subsets. Novel biologics and small molecule inhibitors targeting specific biomarkers and immune signals are much more effective and safer than conventional systemic therapies for these skin diseases. This Research Topic focuses on the interaction between the skin and immune cells and explores how skin cells and immune cells interact with each other and contribute to the pathogenesis of those skin diseases.

The review from Dainichi and Iwata can be considered the grand opening of the Research Topic. The epithelial–immune microenvironment (EIME) of epithelial tissues has five common elements (1): microbial flora (2), barrier (3), epithelial cells (4), immune cells, and (5) peripheral nerve endings. EIME provides both constant defense and situation-specific protective responses through three-layered mechanisms comprising barriers, innate immunity, and acquired immunity. The interactions between the five EIME elements of the skin protect against external dangers from the environment. They show five EIME models: atopic dermatitis, psoriasis, SLE, alopecia areata, and acne to simplify the disease pathologies.

We should know more about epidermal cells to understand the interactions between the skin and other cells. Epidermal keratinocytes can recognize various cytokines and pathogen-associated molecular patterns and produce a wide variety of inflammatory cytokines, chemokines, and antimicrobial peptides. Morizane et al. summarize the foundation of knowledge on the cytokines recognized or produced by epidermal keratinocytes.

The common inflammatory skin diseases atopic dermatitis (AD) and psoriasis are now known as systemic immune skin diseases with Th skewing. Although these two inflammatory diseases often overlap clinically, it has been reported that each can be distinguished by its own genetic biomarkers (1). AD is a Th2-dominant chronic inflammatory skin disease, and acquired immunity plays an important role in the pathogenesis of AD. On the other hand, there is increasing evidence that toll-like receptors (TLRs) are involved in the pathomechanisms of not only infectious diseases, but also non-infectious inflammatory diseases. It has been demonstrated that TLRs recognize both exogenous threats, e.g. bacteria and viruses, and endogenous danger signals related to inflammation, cell necrosis, or tissue damage. Tamagawa-Mineoka review the current understanding of the roles of TLR signaling in the pathogenesis of AD, with particular emphasis on skin barrier function and inflammation.

Recently, Th balances in AD have reported to vary with the age of onset and ethnicity (2, 3). Li et al. investigate that the H2 antigen associated with blood type O is expressed in the granular and horny layers of the skin and plays a protective role in AD-like inflammation.

The factors that can potentially trigger or contribute to AD include genetic factors, family history, dietary choices, immune triggers, and environmental factors (Afshari et al.). Narrow targeting agents blocking IL-4, IL-13, and IL-31 signaling have exhibited significant clinical benefits in patients with AD (4). Stimulation of IL-31 cognate receptors on C-fiber nerve endings believe to activate neurons in the dorsal root ganglion, causing the itch. The IL-31 receptor is a heterodimer of oncostatin M receptor (OSMR) β and IL31RA subunits, and OSMRβ can also bind OSM, a pro-inflammatory cytokine released by monocytes/macrophages, dendritic cells, and T lymphocytes. Suehiro et al. investigate OSM, released from monocytes in the skin, modulates the sensitivity of dorsal root ganglion neurons to Th2 inflammatory cytokines and thereby the severity of AD-associated skin itch. *Staphylococcus aureus* (*S. aureus*) can be frequently found on the skin of AD patients where it actively contributes to skin inflammation. Focken and Schittek describe that *in vitro* co-culture, polymorphonuclear neutrophils (PMNs), and primary human keratinocytes (PHKs) induce inflammatory responses in PHKs which are further exacerbated in the presence of *S. aureus* and induces further PMN recruitment thus fueling skin inflammation. Interestingly, infection of PHKs with the skin commensal *S. epidermidis* reduces the inflammatory effects of PMNs in the skin and has an anti-inflammatory effect. Yu et al. have established a novel mitochondrial-based molecular signature that considers IDH3A, BAX, MRPS6, and GPT2. Their study combines bioinformatics analysis and machine learning to increase their understanding of the crosstalk relationship among these key genes, AD immune infiltration, and mitochondrial metabolic function. In addition, they describe that plasma circulating cell-free mitochondrial DNA may be a key indicator of AD progression, providing evidence of mitochondrial oxidative stress damage during the advancement of AD in adult patients with moderate-to-severe AD.

Psoriasis is associated with various systemic diseases, including cardiovascular disease, diabetes, metabolic syndrome, and several autoimmune diseases. Several biomarkers and signaling pathways are candidates as predictors of cardiovascular disease in patients with psoriasis (5). Clinical studies have suggested a bidirectional association between non-alcoholic steatohepatitis (NASH) and psoriasis, affecting each other’s development and severity. Takezaki et al. investigate the co-occurrence of NASH exacerbate psoriatic skin changes associated with increased serum inflammatory cytokine levels and decreased serum adiponectin levels. They suggest that therapeutic intervention for co-occurring NASH is essential to achieve a favorable prognosis of psoriasis in clinical practice. Kamata and Tada give a general overview of the pathogenesis of psoriasis. Various immune cells are involved in the pathogenesis of psoriasis, including dendritic cells, Th17 cells, and resident memory T cells. Furthermore, keratinocytes play a role in the development of psoriasis as immune cells by secreting antibacterial peptides, chemokines, tumor necrosis factor-α, IL-36, and IL-23. These immune cells and skin cells interact and drive the aberrant differentiation and proliferation of keratinocytes. Among those peptides and chemokines, Liang et al. focus on S100 proteins as potential therapeutic targets and diagnostic biomarkers in psoriasis. Regulatory T cells (Tregs) maintain immune tolerance and prevent autoimmune diseases. They suppress the activation and proliferation of other immune cells, thereby controlling immune responses and reducing inflammation. Tregs diminish during psoriatic inflammation. Lee et al. investigate that treatment with cytotoxic T lymphocyte antigen-4 signaling peptide diminishes psoriatic skin inflammation with increased Treg cell proportion and reduces IL-17 production by T cells.

The crosstalk between the skin and immune cells is important not only in inflammatory skin diseases but also in other skin conditions. Zhang et al. show CD64 play a crucial role in wound healing, especially in diabetes mellitus conditions, where it is associated with CD163^+^ M2 macrophage infiltration. Cuproptosis is a copper-induced cell death reported by Tsvetkov et al. in 2022 (6). Song et al. describe identification of several cuproptosis-related genes as novel therapeutic targets for hypertrophic scar using single-cell analysis and machine learning techniques. Feng et al. discuss the role of macrophages in acne vulgaris. Macrophages embody a paradoxical role in acne development, serving as both sentinels and provocateurs. Their vital functions include the regulation of lipid concentrations and facilitating the elimination of *Cutibacterium acnes*. However, an excessive immune reaction can provoke inflammation and subsequent acne scarring. It is imperative to comprehend their intricate roles to maintain physiological equilibrium and circumvent adverse pathological outcomes. Lu et al. report the case of Sweet syndrome during the lymphoma treatment, with rare clinical presentations of local crater-like suppurative skin lesions. The incidence of Sweet syndrome is high in hematological malignancy but rare in lymphomas.

This Research Topic describes how the skin interacts with immune and other cells, microbes, and nerves, leading to specific inflammation and immune imbalances. The skin and its cells interact with other cells and organs through a “conversation” with cytokines and chemokines, regulating the clinical condition of various skin diseases. A deeper understanding of these interactions in the skin may facilitate the development of targeted therapeutic approaches for skin diseases.
